# P-1651. Long-term Outcomes Compared between Children Hospitalized for Multisystem Inflammatory Syndrome in Children (MIS-C) and Children Hospitalized for Acute COVID-19, New Vaccine Surveillance Network (NVSN) — United States, 2020-2022

**DOI:** 10.1093/ofid/ofaf695.1826

**Published:** 2026-01-11

**Authors:** Anna R Yousaf, Michael J Wu, Brian R Lee, Elizabeth P Schlaudecker, Marian G Michaels, Kristina Betters, Danielle M Zerr, Geoffrey A Weinberg, Leila C Sahni, Jennifer E Schuster, Mary A Staat, John Williams, Laura S Stewart, Eileen J Klein, Peter G Szilagyi, Julie A Boom, Rangaraj Selvarangan, Natasha B Halasa, Janet A Englund, Tiphanie Vogel, Ami B Shah, Ariana Toepfer, Sharon Saydah, Heidi L Moline, Angela P Campbell

**Affiliations:** Centers for Disease Control and Prevention, Atlanta, Georgia; Centers for Disease Control and Prevention, Atlanta, Georgia; Children's Mercy Kansas City, Kansas City, Missouri; Cincinnati Children's Hospital Medical Center, Cincinnati, Ohio; University of Pittsburgh/ CHP, Pittsburgh, Pennsylvania; Vanderbilt University Medical Center, Nashville, Tennessee; University of Washington/Seattle Children's Hospital, Seattle, WA; University of Rochester Sch Med & Dent, Rochester, New York; Baylor College of Medicine and Texas Children's Hospital, Houston, Texas; Children's Mercy Kansas City, Kansas City, Missouri; Cincinnati Children's Hospital Medical Center, Cincinnati, Ohio; University of Wisconsin, Madison, Wisconsin; Vanderbilt University School of Medicine, Nashville, Tennessee; Seattle Children's Hospital and University of Washington School of Medicine, Seatte, Washington; UCLA, Los Angeles, California; Baylor College of Medicine, Houston, Texas; Children’s Mercy Hospital, Kansas City, Missouri; Vanderbilt University Medical Center, Nashville, Tennessee; Seattle Children’s Hospital/Univ. Washington, Seattle, Washington; Texas Children’s Hospital, Houston, Texas; General Dynamics Information Technology, Atlanta, Georgia; Centers for Disease Control and Prevention, Atlanta, Georgia; Centers for Disease Control and Prevention, Atlanta, Georgia; US-CDC, Atlanta, Georgia; Centers for Disease Control and Prevention, Atlanta, Georgia

## Abstract

**Background:**

Few studies compare long-term outcomes in children hospitalized for multisystem inflammatory syndrome in children (MIS-C) with children hospitalized for acute COVID-19. To inform clinical care and resource allocation, we compared patient characteristics and new post-hospitalization symptoms and diagnoses.

**Table 1.**
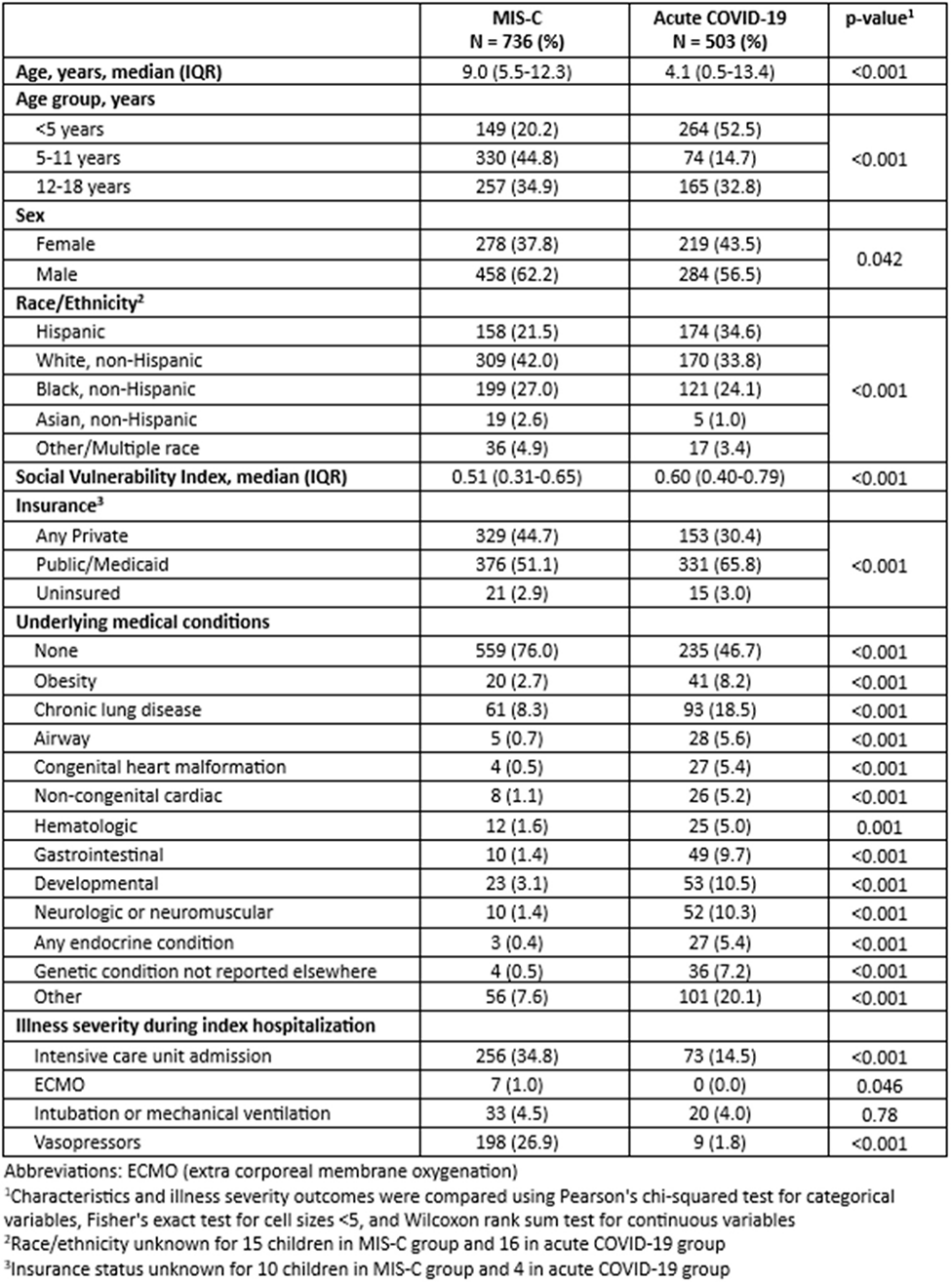
Clinical and demographic characteristics of children hospitalized for MIS-C or acute COVID-19 at time of hospitalization

**Table 2.**
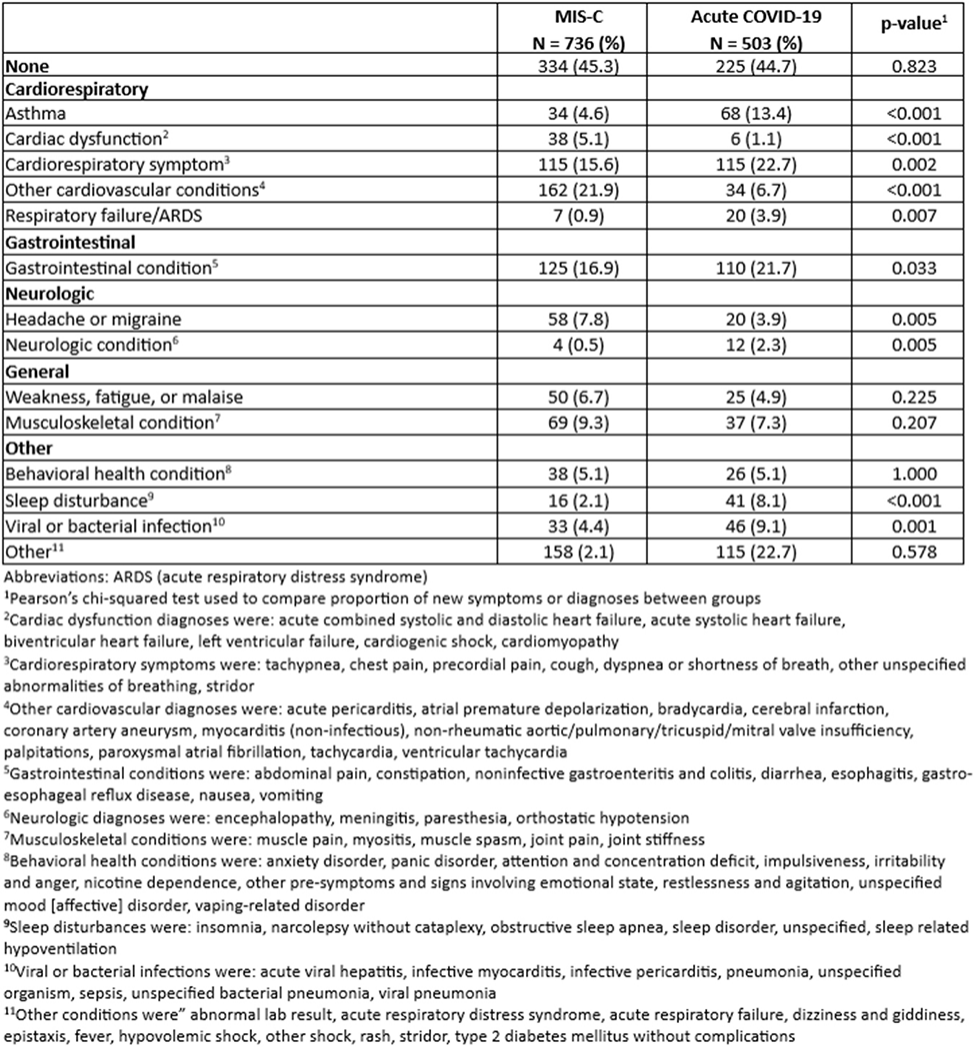
Number of children with new symptoms or diagnoses documented by ICD-10 code in the two years post-hospitalization for MIS-C or acute COVID-19

**Methods:**

Children ≤18 years hospitalized April 2020-April 2022 for MIS-C or COVID-19 at 7 medical centers in the New Vaccine Surveillance Network (NVSN) were included. All children with MIS-C were identified from medical records; children with COVID-19 were limited to those previously enrolled in NVSN. Record abstraction and electronic data extraction were used to collect hospitalization data, and ICD-10 codes of interest within the hospital system in the 2 years before and after hospitalization. “New” symptoms and diagnoses were defined as those with ICD-10 codes not present pre-hospitalization and present post-hospitalization. We used Pearson’s chi-squared, Fisher's exact, and Wilcoxon rank sum tests to assess differences in characteristics and outcomes between MIS-C and COVID-19 patients.

**Results:**

Among 736 children with MIS-C and 503 with COVID-19, children with MIS-C were older (median age 9.0 vs 4.1 years), lived in areas with lower median social vulnerability index scores (0.51 vs 0.60), and more often had no underlying medical conditions (76% vs 47%) (p< 0.001 for all; Table 1). Children with MIS-C more often required ICU admission (35% vs 15%; p< 0.001). After hospitalization, 55% in each group had ≥ 1 new diagnosis or symptom. Children with MIS-C more frequently had cardiac dysfunction (5% vs 1%) or other cardiovascular conditions (22% vs 7%) (p< 0.001 for each; Table 2). Children with COVID-19 more frequently had new diagnoses of asthma (13% vs 5%, p< 0.001), cardiorespiratory symptoms (23% vs 16%, p=0.002), gastrointestinal conditions (22% vs 17%, p=0.033), and sleep disturbances (8% vs 2%, p< 0.001; Table 2).

**Conclusion:**

Children hospitalized for MIS-C and COVID-19 differed by several characteristics including age, underlying conditions, and illness severity. After hospitalization, children with MIS-C more frequently had cardiac conditions; children with COVID-19 more frequently had respiratory, gastrointestinal, and sleep-related symptoms or diagnoses.

**Disclosures:**

Brian R. Lee, PhD, MPH, Merck: Grant/Research Support Elizabeth P. Schlaudecker, MD, MPH, Gilead: Grant/Research Support|Pfizer: Grant/Research Support|Sanofi Pasteur: Advisor/Consultant Marian G. Michaels, MD, MPH, Merck: Grant/Research Support Danielle M. Zerr, MD MPH, AlloVir: Advisor/Consultant|Merck (Any division): Grant/Research Support Geoffrey A. Weinberg, MD, Inhalon Biopharma: Advisor/Consultant|Merck & Co: Honoraria Mary A. Staat, MD, MPH, Centers for Disease Control and Prevention: Grant/Research Support|Cepheid: Grant/Research Support|Merck: Advisor/Consultant|Merck: Grant/Research Support|National Institutes of Health: Grant/Research Support|Up-To-Date: Royalties Rangaraj Selvarangan, PhD, Altona: Grant/Research Support|Biomerieux: Advisor/Consultant|Biomerieux: Grant/Research Support|Biomerieux: Honoraria|Cepheid: Grant/Research Support|Hologic: Grant/Research Support|Hologic: Honoraria|Meridian: Grant/Research Support|Qiagen: Grant/Research Support Natasha B. Halasa, MD, CSL-Seqirus: Advisor/Consultant|Merck: Grant/Research Support Janet A. Englund, MD, AstraZeneca: Board Member|AstraZeneca: Grant/Research Support|Cidarra: Member Data Safety Monitoring Board|GlaxoSmithKline: Advisor/Consultant|GlaxoSmithKline: Grant/Research Support|Meissa Vaccines: Advisor/Consultant|Merck: Advisor/Consultant|Merck: Grant/Research Support|Moderna: Advisor/Consultant|Moderna: Grant/Research Support|Pfizer: Advisor/Consultant|Pfizer: Grant/Research Support|Shionogi: Grant/Research Support Tiphanie Vogel, MD, PhD, AstraZeneca: Grant/Research Support|Moderna: Advisor/Consultant|Pfizer: Advisor/Consultant|SOBI: Advisor/Consultant|SOBI: Board Member|Takeda: Honoraria

